# Occurrence of Pharmaceuticals and Other Anthropogenic Compounds in the Wastewater Effluent of Arctic Expedition Cruise Ships

**DOI:** 10.1021/acs.estlett.5c00209

**Published:** 2025-04-30

**Authors:** Veronica van der Schyff, Marek Stiborek, Zdeněk Šimek, Branislav Vrana, Verena Meraldi, Andrew Luke King, Lisa Melymuk

**Affiliations:** 1 RECETOX, Faculty of Science, Masaryk University, Kotlarska 2, 61137 Brno, Czech Republic; 2 HX (formerly Hurtigruten Expeditions), 210 Pentonville Road, N1 9JY London, U.K.; 3 Norwegian Institute for Water Research (NIVA), Økernveien 94, 0579 Oslo, Norway

**Keywords:** antibiotics, marine pollution, personal care products, blackwater, graywater

## Abstract

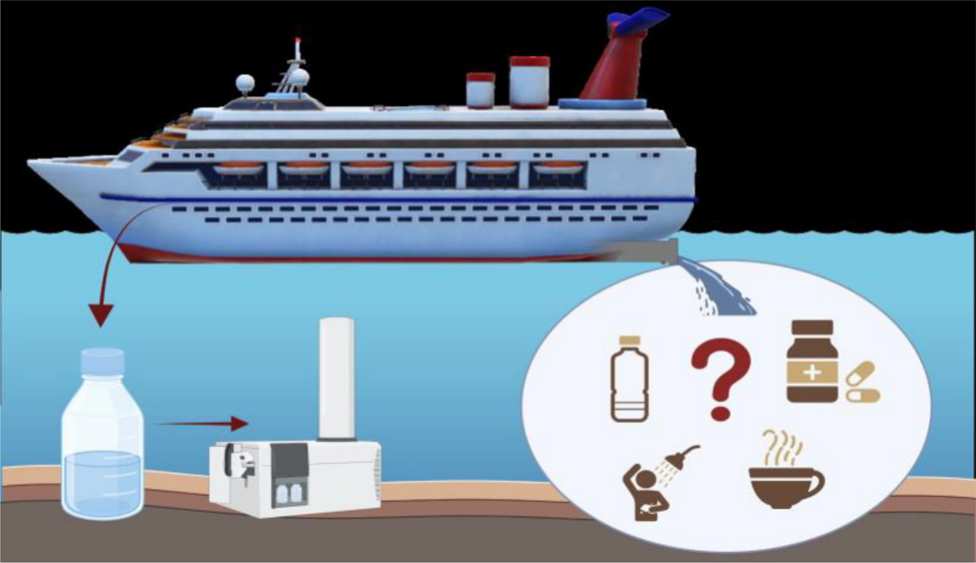

Cruise ship traffic in polar regions is increasing, but we lack a good understanding of the emissions from these ships to sensitive marine environments. Wastewater discharges may result in the release of contaminants of emerging concern into such environments. Treated wastewater from three expedition cruise ships was collected and analyzed with a focus on pharmaceuticals, personal care products, and industrial chemicals. Samples were screened using data-dependent acquisition using liquid chromatography-high-resolution mass spectrometry (LC-HRMS). More than 160 compounds were identified at Schymanski level 1 or 2 (SL1 or SL2, respectively) in treated wastewater across all three ships. Twenty-seven compounds were identified in wastewater from all three ships, suggesting their potential wider presence in ship wastewater and warranting further investigation. For all ships, pharmaceuticals dominated in terms of the number of compounds identified at SL1 or SL2 (43–59%), primarily cardiovascular medications, followed by industrial chemicals (21–31%) and natural compounds (12–17%). Multiple antibiotics were identified at SL1, raising concerns that ship wastewater effluent could contribute to the undesired spread of antibiotic-resistance genes. With the ongoing growth of the cruise industry and uncertainties related to impacts on sensitive marine environments, further investigation of ship wastewater emissions is recommended.

## Introduction

The cruise ship industry is one of the fastest-growing industries in the tourism sector,^1^ with a growth of 7% from 2019 to 2023, and a forecasted growth of 10% from 2024 to 2028.^2^ The size, passenger capacity, and amenities of modern cruise ships make them comparable to terrestrial settlements. As such, they can inflict similar pressures on the surrounding environment, such as noise, air, and water pollution.^3−5^

Due to improvements in ship design and technology, as well as receding polar ice, cruising in polar regions has seen a dramatic increase in recent years.^6^ Traditional and expedition cruises in Arctic regions such as Greenland, Iceland, Svalbard, and northern Norway have increased by 118% between 2018 and 2021.^7^ The Arctic is already under pressure due to climate change, long-range transport of contaminants, and resource exploitation.^8−10^ The increased number of cruise ships presents an additional and understudied stressor and a source of pollution to the region.

Cruise ships discharge wastewater generated from onboard activities. Many ships have advanced wastewater treatment systems (WWTS) onboard. Passenger ships produce graywater (drainage from dishwashers, galley sinks, showers, laundry, baths, and washbasins) and blackwater (from toilets, sanitary areas, and areas with animals). The volume of graywater discharged by cruise ships in 2023 was estimated to be 14 billion L, a 40% increase from 2014 to 2023, largely driven by the growing number of cruise ships.^11^

According to the International Convention for the Prevention of Pollution from Ships (MARPOL) Annex IV,^12^ treated blackwater may be discharged more than 3 nautical miles (NM) from the nearest land. Untreated blackwater may be discharged more than 12 NM from land, as the high seas are considered to be capable of assimilating blackwater through natural bacterial action.^12^ Similarly, the Polar Code states that untreated blackwater should be discharged more than 12 NM from land, fast ice, or ice shelf, and treated wastewater >3 NM.^13^ There are currently no global regulations on the emission of graywater.^14^ Depending on the onboard WWTS, gray- and blackwater can be discharged separately or together. The Baltic Sea is currently the only “special area” under MARPOL Annex IV. There, the discharge of untreated blackwater is strictly prohibited by passenger ships, and where possible, ships are expected to discharge blackwater at ports with reception facilities.^15^

Conventional terrestrial-based WWTS do not eliminate all chemical compounds, and treated effluent contains a complex mix of organic and inorganic micropollutants that are then released into the environment.^16−18^ This limitation likely extends to ship-based WWTS, particularly given their faster processing times and space constraints compared to terrestrial systems. As with the general population, cruise passengers and crew take medication and excrete metabolites into wastewater, with a potential impact on marine environments.^19^ A particular concern is the release of antibiotics, which can promote the development of antibiotic-resistant microbes.^4,5^ In addition, ship wastewater may contain other compounds like surfactants, personal care products, plasticizers, and flame retardants, which are often persistent, bioaccumulative, and toxic to biota.^4^ Ocean currents can transport these contaminants from their point of release to remote areas, potentially affecting sensitive regions and ecosystems. Arctic regions are especially vulnerable to pollution since the efficiency of the naturally occurring bacterial decay of organic contaminants in seawater is slowed by low temperatures. Other environmental factors such as sea ice and fluctuating ultraviolet light exposure due to large seasonal variation also impact pollutant accumulation patterns in the Arctic.^20,21^

With the number and passenger capacity of cruises increasing, it is unclear whether dilution remains sufficient to mitigate the impact of contaminants released from wastewater, particularly in regions with a high density of cruise ships. Pharmaceuticals and other chemicals have been detected in seawater in remote Arctic regions.^20,22^ While substandard WWTS in remote Arctic regions are considered the main source of contaminants,^23^ other studies^24,25^ suggest that the effects of ship effluent should also be studied as a source of pollution.

Limited attention has been paid to wastewater effluent discharges of cruise ships. To the best of our knowledge, only two studies have quantified synthetic compounds in treated wastewater,^4,26^ and no chemical screening has been conducted on treated wastewater intended for offshore discharge from cruise ships while in operation. This study aims to identify organic contaminants associated with wastewater discharge from cruise ships as a first step to better evaluate the impact of contaminant emissions from cruise ships on sensitive marine environments.

## Methods and Materials

### Ship Description

Wastewater samples were collected from three expedition cruise ships with varying operational ages and conditions. Ship 1 (launched 2002, renovated 2020, 530 passengers, 16 000 Gross tonnage (GT)), ship 2 (launched 2003, 570 passengers, 16 000 GT), and ship 3 (launched 2020, 530 passengers, 21 000 GT) all frequently operate in the North Atlantic Ocean and often travel above the Arctic Circle (66°30′ N). The passenger demographics on all three ships during the sampling cruises were predominantly retired individuals.

Ships 1 and 3 feature an advanced wastewater treatment purification system (AWP) combining biological treatment, multistage filtration, and sludge processing for efficient black- and graywater treatment. The WWTS on board ship 1 was retrofitted during renovation, while the system was built into ship 3 during construction. Ship 2 uses a physiochemical wastewater treatment system based on air flotation, chemical coagulation, and UV disinfection. The system is primarily designed for blackwater treatment but can function with a mixture of blackwater and graywater.

### Sample Collection

Sampling occurred once while each ship was at sea, shortly before wastewater discharge >3 NM offshore, to ensure the collected samples represented peak operational conditions. Two liters of wastewater were collected in polycarbonate Nalgene bottles from each ship’s final holding tank, containing a mixture of treated black- and graywater ready for offshore discharge. Samples were then filtered through preconditioned AttractSPE Disks HLB (Affinisep, France, Hydrophilic/Lipophilic Balanced; Supporting Information) disks in aliquots of 100–150 mL onboard. Although aliquot volumes varied, this is not a limitation as the study focused on qualitative suspect screening rather than quantitative concentration measurements. After filtration, samples were dried by mild underpressure, stored chilled at 4 °C in the ship laboratory refrigerator for 7 days, transported to the laboratory in the Czech Republic in cooling bags with ice packs, and subsequently frozen at −20 °C for storage at the Trace Laboratory of RECETOX, Czech Republic, until extraction.

### Sample Extraction and Cleanup

Immediately prior to sample processing, HLB disks were transferred to a −80 °C freezer in preparation for freeze-drying and subsequently freeze-dried. An extraction protocol adapted from Moschet et al.^27^ was utilized to isolate analytes. The disks underwent sequential extraction with acetone and methanol, after which the combined solvent fractions were concentrated to 1 mL by using a mild nitrogen stream. The final extract was filtered and prepared for analysis via liquid chromatography coupled to high-resolution mass spectrometry (LC-HRMS). A detailed description is provided in the Supporting Information.

### LC-HRMS Analyses and Data Processing

In brief, high-resolution mass spectrometry was used for qualitative analyses of the wastewater extracts. Two aliquots per ship were analyzed. Chromatographic separation was performed using an Agilent 1290 Infinity LC System (Agilent Technologies, Santa Clara, CA) equipped with a Kinetex Core–Shell Biphenyl column (150 mm × 2.1 mm, particle size of 1.7 μm) and a precolumn (Phenomenex, Torrance, CA). Mass spectrometry detection was performed by using an Agilent 6550 Q-TOF iFunnel System operating in positive and negative electrospray ionization (ESI) modes. Iterative data-dependent acquisition (DDA) of MS/MS spectra was applied, with a maximum of four precursor ions fragmented per cycle at collision energies of 10, 20, and 40 eV. A detailed description of the analytical method is presented in the Supporting Information.

Combined full-scan and MS/MS raw data files were processed with Agilent MassHunter Qualitative Analysis Software 10.0 (Agilent Technologies). Data processing included peak picking, molecular formula assignment, isotope pattern scoring, and identification of the found features based on compliance with the predicted molecular formula and MS/MS spectra present in mass spectrum libraries. A full list of databases used and quality assurance is included in the Supporting Information. All compounds reported here were identified at confidence level 1 or 2 according to the Schymanski identification confidence levels (SL1, confirmed a with reference standard, or SL2, probable structure; library match or diagnostic evidence) in high-resolution mass spectrometric analysis.^28^

## Results and Discussion

**Figure 1:**
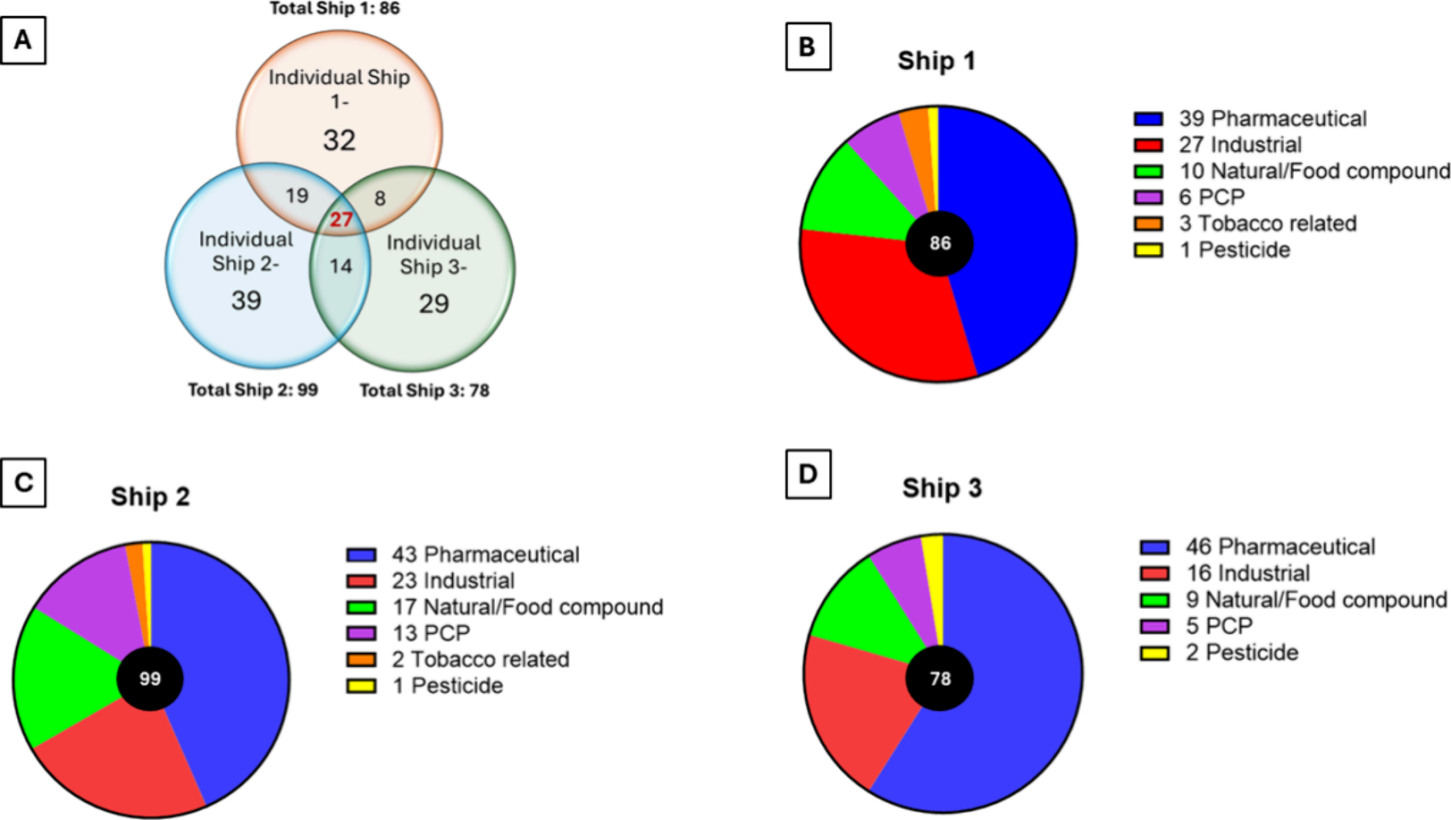
(A) Number of compounds identified at SL1 or SL2, in three expedition ships showing overlap in the compounds found in wastewater among the three ships and (B–D) distribution and number of confirmed or probable compounds according to use category in wastewater effluent from three expedition ships.

Wastewater samples from the older ships built in the 2000s (ships 1 and 2) had >11 000 found features, while the newest ship, ship 3, built 2020, had 7571 (Table S1). From these features, a total of 168 compounds were confirmed at SL1 or the chemical structure was proposed at SL2 in treated wastewater across all three ships: 86, 99, and 78 compounds from ships 1–3, respectively (Figure 1 and Tables S2–S4). Twenty-seven compounds were identified at SL1 or SL2 across all ships (Table 1), with pharmaceuticals and industrial chemicals (10 each) constituting the majority, followed by five food/natural compounds, one pesticide, and one UV blocker. Eight of the 27 compounds common to all ships were identified at SL1 based on external standards (EnviMix, Helmholtz-Centre for Environmental Research^29^), and 19 identified at SL2.

**Table 1:** Compounds Identified at SL1 or SL2 in Wastewater from All Ships^a^

compound	CAS Registry No.	category	usage	usage source^b^
caffeine*	58-08-2	food compound	stimulant	T3DB
harman	486-84-0	food compound	formed in grilled food items and tobacco smoke	CompTox
choline	62-49-7	natural/food compound	essential vitamin	FooDB
indole	120-72-9	natural	food component and flavoring ingredient	FooDB
thiamine thiozole	137-00-8	natural/food compound	flavoring agent	PubChem
triphenylphosphine oxide*	791-28-6	industrial	catalyst	PubChem
1-naphthylsulfonic acid	85-47-2	industrial	colorant mainly used in dye production	CompTox
4-isopropylbenzenesulfonic acid	16066-35-6	industrial	plating agent and surfactant	PubChem
aniline	62-53-3	industrial	solvent, chemical and intermediate for dye, polymer, and rubber industries	PubChem
denatonium*	47324-98-1	industrial	deterrent added to toxic household products and chemicals to prevent ingestion	PubChem
diphenyl phosphate	838-85-7	industrial	flame retardant, metabolite of TPHP	CompTox
tris(2,4-di-*tert*-butylphenyl) phosphate*	95906-11-9	industrial	polymer processing aid	PubChem
edetol	102-60-3	industrial/PCP	industrial: multiple uses, including as a chelating agent and catalyst; cosmetic, chelating agent	PubChem
stearamide (octadecanamide)	124-26-5	industrial/PCP	industrial, lubricant; cosmetic, foam boosting	PubChem
cyclamic acid	100-88-9	industrial/food compound	paint and plastic production, compound salt (cyclamate) used as an artificial sweetener	CompTox
				
8-chlorotheophylline	85-18-7	pharmaceutical	stimulant	DrugBank
atenolol acid (metoprolol acid)	56392-14-4	pharmaceutical	β-blocker	PubChem
bisoprolol	66722-44-9	pharmaceutical	β-blocker and treatment of hypertension	DrugBank
candesartan	139481-59-7	pharmaceutical	hypertension treatment	DrugBank
irbesartan	138402-11-6	pharmaceutical	treats hypertension and diabetic kidney issues	DrugBank
losartan*	114798-26-4	pharmaceutical	treats hypertension, reduces diabetic kidney issues, and decreases stroke risk	DrugBank
telmisartan	144701-48-4	pharmaceutical	treats hypertension and heart failure	DrugBank
flecainide	54143-55-4	pharmaceutical	antiarrhythmic agent	DrugBank
diphenhydramine*	58-73-1	pharmaceutical	antihistamine	DrugBank
fexofenadine*	83799-24-0	pharmaceutical	antihistamine	DrugBank
				
phenylbenzimidazole sulfonic acid	27503-81-7	PCP	UV filter	EU CosIng
				
DEET/diethyltoluamide*	134-62-3	pesticide	insect repellent	DrugBank

a An asterisk indicates compounds that were confirmed by external standards. Compounds with multiple uses in different categories were categorized by the most probable use, but with the secondary category noted.

b Compound uses were obtained from T3DB,^30^ Comptox,^31^ FooDB,^32^ PubChem,^33^ DrugBank,^34^ and EU CosIng.^35^

For all ships, pharmaceuticals, primarily cardiovascular medication, dominated in terms of the number of compounds identified at SL1 or SL2 (43–59%), followed by industrial chemicals (21–31%) and natural/food compounds (12–17%) (Figure 1).

Industrial chemicals such as surfactants, lubricants, and cleaning products can originate from onboard operations, or from being incorporated into onboard products, like in the case of flame retardants and plasticizers, with release to dust and transfer to wastewater during cleaning or laundering.^36^ Many natural compounds can originate from food preparation and consumption, including some with potential environmental impacts, such as caffeine and aromatic amines, the latter being mutagenic.^37^

The compound group of the highest concern consists of pharmaceuticals due to their potential biological activity at low concentrations.^38^ The pharmaceuticals were primarily cardiovascular medications, with the sartan group (losartan, irbesartan, candesartan, and telmisartan) identified at SL1 or SL2 (Table 1) in all three ship wastewater profiles. Other cardiovascular medications, flecainide, and bisoprolol, were also identified in wastewater from all three ships (Table 1).^25^ Since the aforementioned medications are persistent and known for incomplete removal, even in typical WWTS, it stands to reason that they should be prominent in wastewater from ships too.^39,40^

Additionally, multiple antibiotics were identified SL1 or SL2 (Tables S2–S4), including sulfamethoxazole, clarithromycin, levofloxacin, and trimethoprim, raising concerns that cruise ship wastewater effluent could contribute to the spread of antibiotic-resistant bacteria and their resistance genes.^4^ According to Chia et al.,^41^ antibiotics are the pharmaceuticals with the most detrimental effects on phytoplankton species, followed by selective serotonin reuptake inhibitors (SSRIs), such as venlafaxine, which was identified at SL2 in wastewater from ships 2 and 3 (Tables S2 and S3). Nonsteroidal anti-inflammatory drugs (NSAIDs), β-blockers, antihistamines, lipid regulators, analgesics, and antiepileptic medicines are less harmful to phytoplankton,^41^ but may have other negative ecosystem impacts. Duarte et al.^42^ found that the NSAID diclofenac and the antibiotic sulfamethoxazole negatively impacted phytoplankton species diversity in freshwater environments and may also affect marine ecosystems. The presence of pharmaceuticals and industrial chemicals in Arctic zooplankton might suggest that bioaccumulation poses broader ecological risks.^25^

There are numerous records of plastic additives and other industrial chemicals in Arctic environments.^25,43−45^ While the presence of such compounds of emerging concern in Arctic systems is typically attributed to long-range transport from temperate zones, the ability to distinguish between local and long-range sources remains a crucial gap.^46^ Recent evidence has highlighted the increasing contribution of local sources as commercialization in Arctic environments increases.^47^ A better understanding of the profile of chemicals originating in ship wastewater can distinguish local sources from long-range transport. However, a majority of data on anthropogenic compounds in Arctic surface water are from the vicinity of inhabited areas,^20,48,49^ where the influence of terrestrial wastewater sources makes it difficult to estimate a baseline for contamination in the Arctic Ocean, and thus difficult to contextualize the input of ship wastewater to the contamination load. Additionally, medium-distance transport of compounds (tens to hundreds of kilometers) from both ships and terrestrial settlements may complicate the process of identifying contamination sources in the Arctic.^50^

Direct comparison between results obtained in this study and previous studies remains challenging due to differences in methodology. Most existing literature on contaminants from ship wastewater has employed targeted analyses that focused on a limited number of compounds, whereas the present study has confirmed multiple compounds without quantification. Vicente-Cera et al.^4^ collected wastewater samples while a ship was docked for repairs, during which the crew was living onboard, though the vessel was not operating at full capacity. Similar concentrations of pharmaceuticals were found in ship wastewater as in urban treatment plants.^4^ Westhof et al.^26^ examined 21 compounds in blackwater and graywater from cruise ships with capacities of 2600–3300 passengers. They found that pharmaceuticals were predominantly present in blackwater, while PCPs, UV filters, and industrial chemicals were more common in graywater. Notably, 13 of the 16 quantified compounds were found in the plant permeate, i.e., the final stage of wastewater treatment prior to effluent discharge, where our samples were also collected.^26^

The average size of cruise ships (including expedition ships) in the North Atlantic region varies, ranging from tens of people to more than 5000 (Figure S1).^51^ In winter, smaller ships with capacities of <1000 are prevalent (Figure S1).^51^ Expedition ships visiting remote polar regions typically carry fewer than 200 people, since remote regions often have restrictions on the number of visitors allowed on land at a time.^52^ However, larger cruise ships often frequent the Norwegian coast, even above the Arctic Circle in cities like Tromsø and Alta and the Norwegian fjords.^53^ Given the relation between populations served and chemical loadings observed in terrestrially based WWTS,^54^ it is expected that the quantities of effluent discharge will scale with ship size, capacity, and cruise duration.

In the North Atlantic, cruise ships built in the 1990s and 2000s are prevalent (Figure S2);^51^ however, information about the type, age, and efficacy of onboard WWTS is not available from maritime databases. This complicates the assessment of broader cruise industry impacts, as the treatment technology onboard can have an impact on the chemical composition of the wastewater effluent. Ship 2, the oldest unrenovated vessel with an industry-standard WWTS, had the highest number of confirmed or proposed compounds, 99 (Figure 1). Ships 1 and 3 had the same advanced WWTS; however, ship 1 was retrofitted with the WWTS, whereas ship 3 had it integrated during construction, which can result in better system performance and may explain the difference in the chemical profiles of the treated wastewater (86 features in ship 1 vs 78 in ship 3; Figure 1).

Other ships, such as naval vessels and cargo ships, are expected to have distinctly different chemical emission profiles. For example, industrial chemicals likely dominate cargo ship wastewater, which have crew sizes that are relatively small compared to ship size, while passenger-heavy ships such as ferries are expected to have similar profiles to cruise ships. The discharge of bilge water and ballast water will also have characteristics distinctly different from those of wastewater, which has not been considered in this screening and warrants investigation. Additionally, other environmental factors at the time of emission, such as the presence of sea ice may affect the behavior, persistence, and distribution of contaminants.^50^

There is a significant gap in our understanding of the types of compounds present in ship wastewater discharges. This issue is becoming increasingly urgent as the cruise industry expands, especially in sensitive polar regions, and as sustainable development and tourism become more important priorities. Identification and quantification of pharmaceuticals and industrial compounds can support better environmental risk assessment. Water in shipping lanes and cruising routes should be studied to evaluate the effectiveness of dilution and degradability in marine water, particularly in areas where ships are permitted to discharge wastewater. If needed, sensitive regions should be denoted (in addition to the 3 and 12 NM limits) where wastewater discharges should be avoided. Special emphasis should be placed on compounds consistently identified across multiple vessels, including SSRIs, antibiotics, and other substances that could pose environmental concerns at increased concentrations. Moreover, the mixture effects of the various chemicals found in wastewater should be thoroughly examined, as the combined presence of different pharmaceuticals and industrial chemicals may result in additive or synergistic effects that can intensify their harmful impact on marine ecosystems.^55,56^ Current regulations and treatment standards of graywater may need reevaluation as we gain an understanding of the impact of trace organic contaminants on marine systems.

## Supporting Information Available

Additional experimental details and methods, data processing and analyses, quality assurance and control, and supplemental figures from data procured from Marine Traffic Database (PDF)
Supplemental tables with total features found in wastewater and specific compounds found in each ship water sample (XLSX)
