# Supplementary material for: Occurrence of Pharmaceuticals and Other Anthropogenic Compounds in the Wastewater Effluent of Arctic Expedition Cruise Ships
Source: Environ Sci Technol Lett. 2025 Apr 30;12(5):648–54. doi: 10.1021/acs.estlett.5c00209 (PMC12080242; doi:10.1021/acs.estlett.5c00209)
Supplement: Supplementary file 1 — ez5c00209_si_001.pdf [file ez5c00209_si_001.pdf]

Occurrence of pharmaceuticals and other anthropogenic compounds in the wastewater effluent of Arctic expedition cruise ships

Veronica van der Schyff[1]*, Marek Stiborek[1], Zdeněk Šimek[1] Branislav Vrana[1], Verena Meraldi[2], Andrew Luke King[3], Lisa Melymuk[1]

* Corresponding author: Veronica van der Schyff. veronica.vanderschyff@recetox.muni.cz

[1]RECETOX, Faculty of Science, Masaryk University, Kotlarska 2, 61137 Brno, Czech Republic

[2]HX (formerly Hurtigruten Expeditions), 210 Pentonville Road, N1 9JY, London, UK

[3]Norwegian Institute for Water Research (NIVA), Økernveien 94, 0579, Oslo, Norway

Supplemental Information: Methods

HLB disks conditioning

Before sampling, HLB solid phase extraction disks (Affinisep SPE, 47 mm) were activated by submerging them in acetone, isopropanol, methanol, and Milli-Q water for 24 hours per solvent.

Sample extraction and cleanup

The HLB disks were frozen at -80°C and freeze-dried to dryness. The freeze-dried disks were extracted using an adapted method based on Moschet et al. (2015). Initially, 10 ml of acetone was added to the vials containing the disks and shaken for 2 hours on a rotary shaker. The acetone fraction was then transferred into a separate vial. This process was repeated with a second 10 ml portion of acetone, and the combined acetone fractions were collected. Subsequently, 10 ml of methanol was added to the disks and shaken for 2 hours.

The 20 ml acetone fraction was evaporated to 1 ml using a nitrogen evaporator. The methanol fraction was then added to the evaporated acetone fraction, and the total mixture was evaporated to 1 ml. Precisely 1 ml (0.79 g) of the extract was weighed out and filtered using a syringe filter and transferred to a pre-weighed 2 ml amber vial.

Liquid chromatography high-resolution mass spectrometry (LC-HRMS) analyses

A chromatographic separation was performed using an Agilent 1290 Infinity LC System (Agilent Technologies, Santa Clara, CA) equipped with a reversed-phase column (Kinetex Core-Shell Biphenyl ,150 × 2.1 mm, particle size 1.7 μm) with a pre-column (both from Phenomenex, Torrance, CA). For the ESI-positive acquisition mode, water (A) and acetonitrile (B), both containing 0.1% v/v formic acid were used as a mobile phase component. In ESI-negative acquisition mode, water (A) and acetonitrile (B), both containing 0.005% acetic acid, were employed. The mobile phase gradient for both ESI-positive and ESI-negative mode was as follows: starting at 10% B at 0 minutes, increasing from 10% B to 98% B over 0-25 minutes, held at 98% B from 25-30 minutes, and then decreasing back to 10% B from 30-31 minutes, followed by an equilibration step at 10% B until 40 minutes. The flow rate was maintained at 0.2 mL/min with the column temperature of 25°C. The injection volume was 5 μL.

A mass spectrometry detection was carried out using an Agilent 6550 Q-TOF iFunnel System (Agilent Technologies, Santa Clara, CA) operated in both ESI-positive and ESI-negative modes. Automatic MS/MS mode with iterative sample analysis for data-dependent acquisition (DDA) was used for acquisition. Each sample was analysed in four iterations. Blank samples were analysed in one iteration due to low matrix expected. In each iteration, a maximum of 4 precursor ions were fragmented per cycle. Mass error tolerance for iterative MS/MS was ±20 ppm and retention time exclusion tolerance was ±0.05 min. The threshold for precursor ions fragmentation was set on 10,000 counts to give quality MS/MS spectra. The fragmentation was performed with a collision energy of 10, 20 a 40 eV. The isolation width for precursor ions was 1.3 *m/z*. The acquisition rate was set on 4 spectra/s for both ESI modes. The parameters of the ion source for ESI-positive mode were: gas temperature 250°C, gas flow 16 L/min, nebulizer pressure 40 psi, sheath gas temperature 400°C, sheath gas flow 12 L/min, capillary voltage 3000 V, and nozzle voltage 0 V. The MS and MS/MS mass ranges were set at 90-1 000 m/z and 40-800 m/z, respectively. The parameters of the ion source for ESI-negative mode were: gas temperature 200°C, gas flow 12 L/min, nebulizer pressure 40 psi, sheath gas temperature 400°C, sheath gas flow 12 L/min, capillary voltage 3000 V, and nozzle voltage 2000 V. The MS and MS/MS mass ranges were 90-1100 m/z and 40-900 m/z, respectively.

Data processing and analysis

Combined full-scan and MS/MS raw data files were processed by Agilent Mass Hunter Qualitative Analysis Software 10.0. Data processing included peak picking, molecular formula assignment, isotope pattern scoring, and identification of the found features based on compliance with the predicted molecular formula and MS/MS spectra present in available libraries. As a source of MS/MS spectra, a commercial Agilent Applied Markets PCDL and open-source Mass Bank EU and Mass Bank North America MS/MS libraries were used. Based on NORMAN SusDat database, Pharmaceuticals (PHARMA), Plant protection products (PPP), Drugs of abuse (DOA), Surfactants (SURF) and Per- and polyfluoroalkyl substances (PFAS) five individual suspect lists were created from above mentioned open sources and used together with Agilent Applied Market PCDL library for final suspect screening. ChemVista Library Manger 1.0 (Agilent) was employed to link the mentioned suspect lists with MS/MS spectra. The mass spectrometer was tuned according to Agilent Tuning Guide for measurement in High Resolution mode achieving a TOF mass resolution of up to 40,000. Agilent ESI-Low Concentration Tuning Mix (G1969-85000) was used for these purposes.

Quality assurance and quality control

Quality assurance and quality control (QA/QC) measures were implemented throughout sample preparation and instrumental analysis to ensure reliable results. Laboratory blank samples (150 ml MilliQ water) were prepared and analyzed alongside each set of individual ship wastewater sample extracts to identify and exclude potential background contaminants. Each sample and blank was analyzed in duplicate to enhance reliability and detect any inconsistencies.

Before instrumental analysis of each set of samples, the mass spectrometer was calibrated using the Agilent ESI-Low Concentration Tuning Mix (G1969-85000), For continuous mass accuracy correction during analysis, an Agilent LC/MS reference mass solution, prepared from the Agilent LC/MS Reference Mass Standard Kit (G1969-85003), was continuously nebulized into the ESI source along with the chromatographic effluent. Background contaminants identified in blank samples were excluded from assessment by comparison with sample extracts.

Supplemental Tables

Table S1. Total features found in wastewater (WW) samples and number of features identified at SL1 or SL2.

	Ship 1 sample 1	Ship 1 sample 2		Ship 2 sample 1	Ship 2 sample 2		Ship 3 sample 1	Ship 3 sample 2
Number of found features in ESI +	7549	8287		7605	7384		4980	3899
Number of found features in ESI -	3581	4494		3801	3870		2591	2873
Total	**11130**	**12781**		**11406**	**11254**		**7571**	**6772**
	Ship 1			Ship 2			Ship 3	
Identified at SL1 or SL2	86			99			78	
Identified at SL1 or SL2 in all ship WW	27							

Tables S2-S4 (Table S2: Ship 1; Table S3: Ship 2; Table S4: Ship 3) in Excel Workbook. Compounds identified at SL1 and SL2 in wastewater from all three ships.

Supplemental Figures

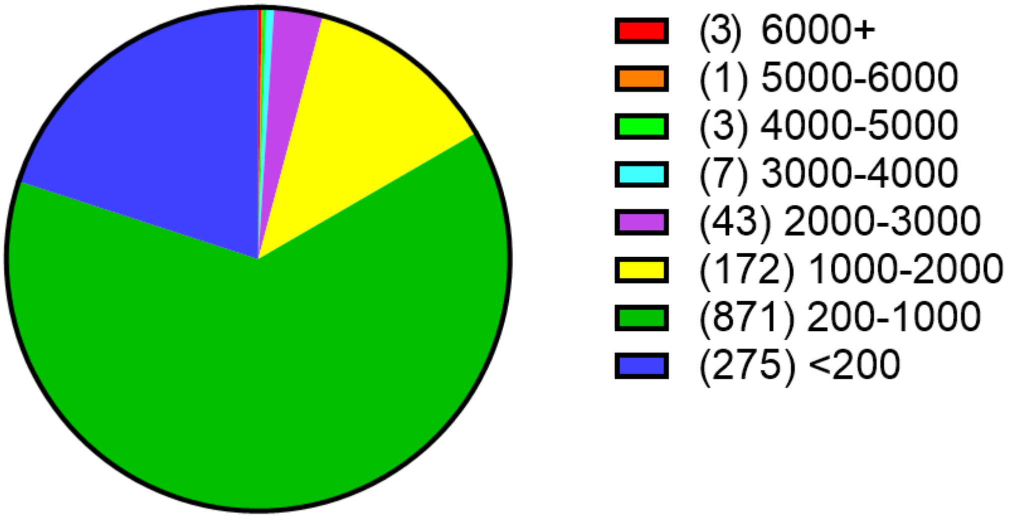

Figure S1. Passenger ships in the North Atlantic as recorded by Marine Traffic Database[1] on 27 December 2023 per passenger capacity. Numbers in parentheses indicate the number of ships. The numbers outside indicate the ship passenger capacity.

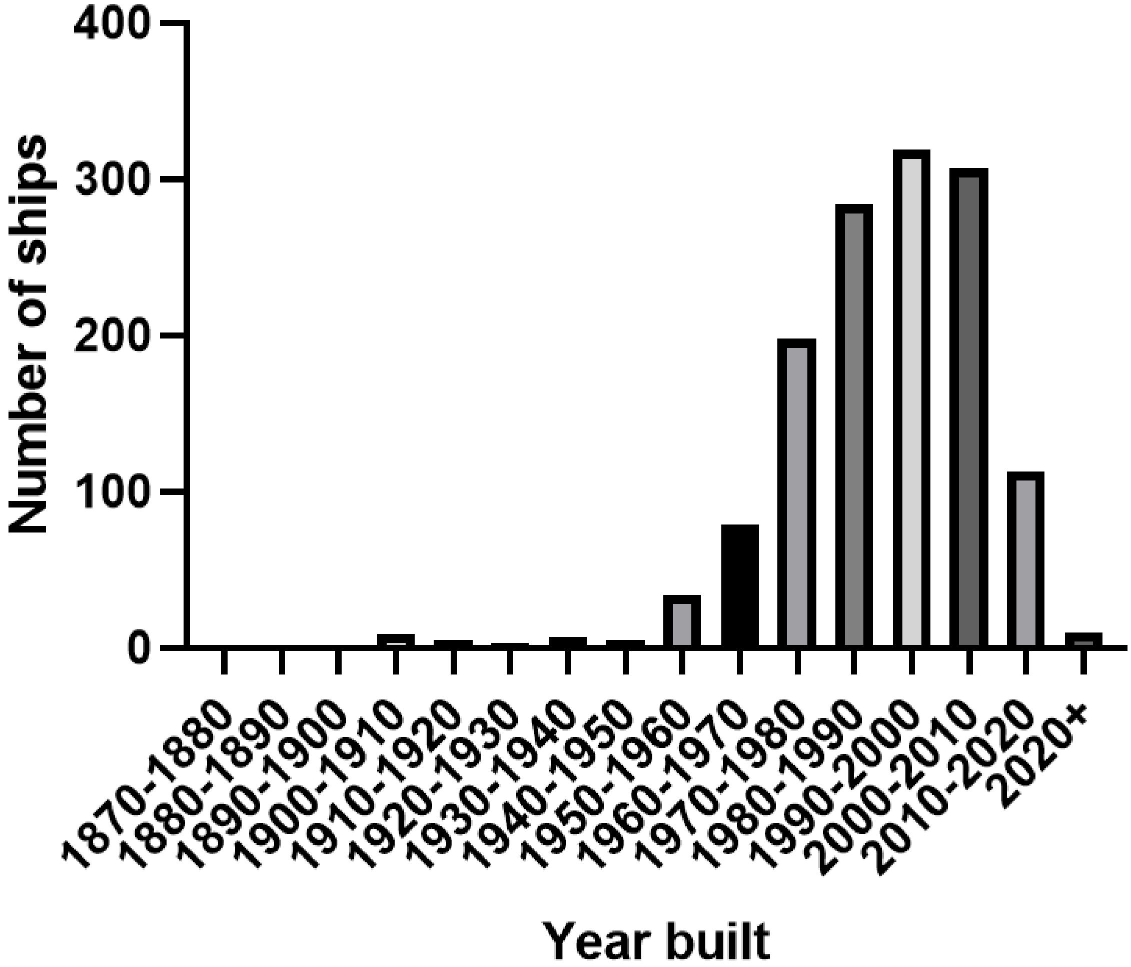

Figure S2. Passenger ships in the North Atlantic as recorded by Marine Traffic Database on 27 December 2023 per ship size.

References

(1) Marine Traffic Data. *Vessels*. https://www.marinetraffic.com/en/data/ (accessed 2023-12-27).
